# Loss of CYLD accelerates melanoma development and progression in the Tg(*Grm1*) melanoma mouse model

**DOI:** 10.1038/s41389-019-0169-4

**Published:** 2019-10-07

**Authors:** Miriam Martha de Jel, Mandy Schott, Susanne Lamm, Winfried Neuhuber, Silke Kuphal, Anja-Katrin Bosserhoff

**Affiliations:** 1 Institute for Biochemistry, University of Erlangen-Nürnberg (FAU), Erlangen, Germany; 2 Institute for Anatomy, University of Erlangen-Nürnberg (FAU), Erlangen, Germany

**Keywords:** Melanoma, Cell growth, Cancer microenvironment, Melanoma, Cell growth

## Abstract

The deubiquitinase cylindromatosis (CYLD) is a well-known tumor suppressor, found to be down regulated in many cancer types including breast cancer, colon carcinoma and malignant melanoma. CYLD is suppressed in human melanoma cells by the transcriptional repressor SNAIL1 leading to an increase of their proliferative, invasive and migratory potential. To gain additional insights into the distinct function of this tumor suppressor gene a new mouse model Tg(*Grm1*)*Cyld*^*−/−*^ was generated. Herewith, we demonstrate that *Cyld*-deficiency leads to earlier melanoma onset and accelerated tumor growth and metastasis in the GRM1 melanoma mouse model. First, RNA sequencing data revealed a potential role of CYLD in the regulation of genes involved in proliferation, migration and angiogenesis. Experiments using cell lines generated from both primary and metastatic melanoma tissue of Tg(*Grm1*) *Cyld*^*−/−*^ and Tg(*Grm1*) *Cyld*^*+/+*^ mice confirmed that loss of CYLD enhances the proliferative and migratory potential, as well as the clonogenicity in vitro. Moreover, we could show that *Cyld*-knockout leads to increased vasculogenic mimicry and enhanced (lymph-) angiogenesis shown by tube formation assays, immunohistochemistry and mRNA expression analyses. In summary, our findings reveal new functional aspects of CYLD in the process of (lymph-) angiogenesis and demonstrate its importance in the early process of melanoma progression.

## Introduction

The tumor suppressor function of cylindromatosis (CYLD) was first described in the disease of familiar cylindromatosis, a rare benign tumor of skin appendages^1^. Since then, both depletion and mutation of CYLD have been associated with development and progression of a variety of tumors such as cancer of the breast, colon and lung^2–4^. CYLD was also shown to be suppressed on mRNA, as well as protein level in human cell lines and tissue samples of malignant melanoma^5–7^. Melanoma represents the most aggressive form of skin cancer with still increasing incidence rates. Here, CYLD expression is down regulated in consequence of elevated SNAIL1 expression, resulting in augmented CyclinD1 and N-Cadherin levels^5^ and in an increase in proliferation, migration and invasion of human melanoma cells. Furthermore, CYLD expression was shown to be inversely correlated with overall and progression-free survival in melanoma patients^5^. On a molecular level, the deubiquitinase CYLD regulates several important signaling pathways as the NF-κB, JNK and Wnt/β-Catenin pathway^8–10^.

In this study, we analyzed the role of CYLD for the first time in a murine model for spontaneous melanoma development. Tg(*Grm1*) mice display pigmented lesions after a short latency and with complete penetrance^11^. GRM1 is a seven transmembrane domain comprising G-protein coupled receptor whose expression was shown to be up-regulated in human melanoma cell lines and tissues compared with melanocytes and normal tissue^11–13^. Since *Grm1* is placed under the control of the melanocyte-specific *Dct* promoter in the Tg(*Grm1*) mouse model, *Grm1* is specifically overexpressed in cells of melanocytic origin leading to cutaneous as well as uveal melanoma^11,14^. Further, in this mouse model the development of metastasis in lymph nodes, lung and liver were observed^15^.

Both angiogenesis and lymph angiogenesis, the formation of new blood or lymphatic vessels from pre-existing ones, are described as crucial processes in melanoma development and progression. It was shown that the induction of angiogenesis can be mediated by single transformed melanoma cells^16^. Further, the correlation between lymph angiogenesis and melanoma progression to distant metastases was described previously^17,18^.

In this report, we demonstrate that *Cyld*-deficiency promotes melanoma onset and growth in the Tg(*Grm1*) mouse model. Furthermore, CYLD has an impact on cellular processes in vitro as it negatively regulates proliferation, migration and colony formation. In addition, we present data that indicate a new regulatory role of CYLD regarding vasculogenic mimicry and (lymph-) angiogenesis in malignant melanoma. Thus, our results confirm the Tg(Grm1) mouse model as a reliable model for further investigations regarding molecular processes in malignant melanoma. Finally, our new findings constitute a step forward in understanding the specific role of the tumor suppressor CYLD in malignant melanoma.

## Results

### Loss of CYLD accelerates melanoma onset and increases tumor growth in vivo

**Fig. 1:**
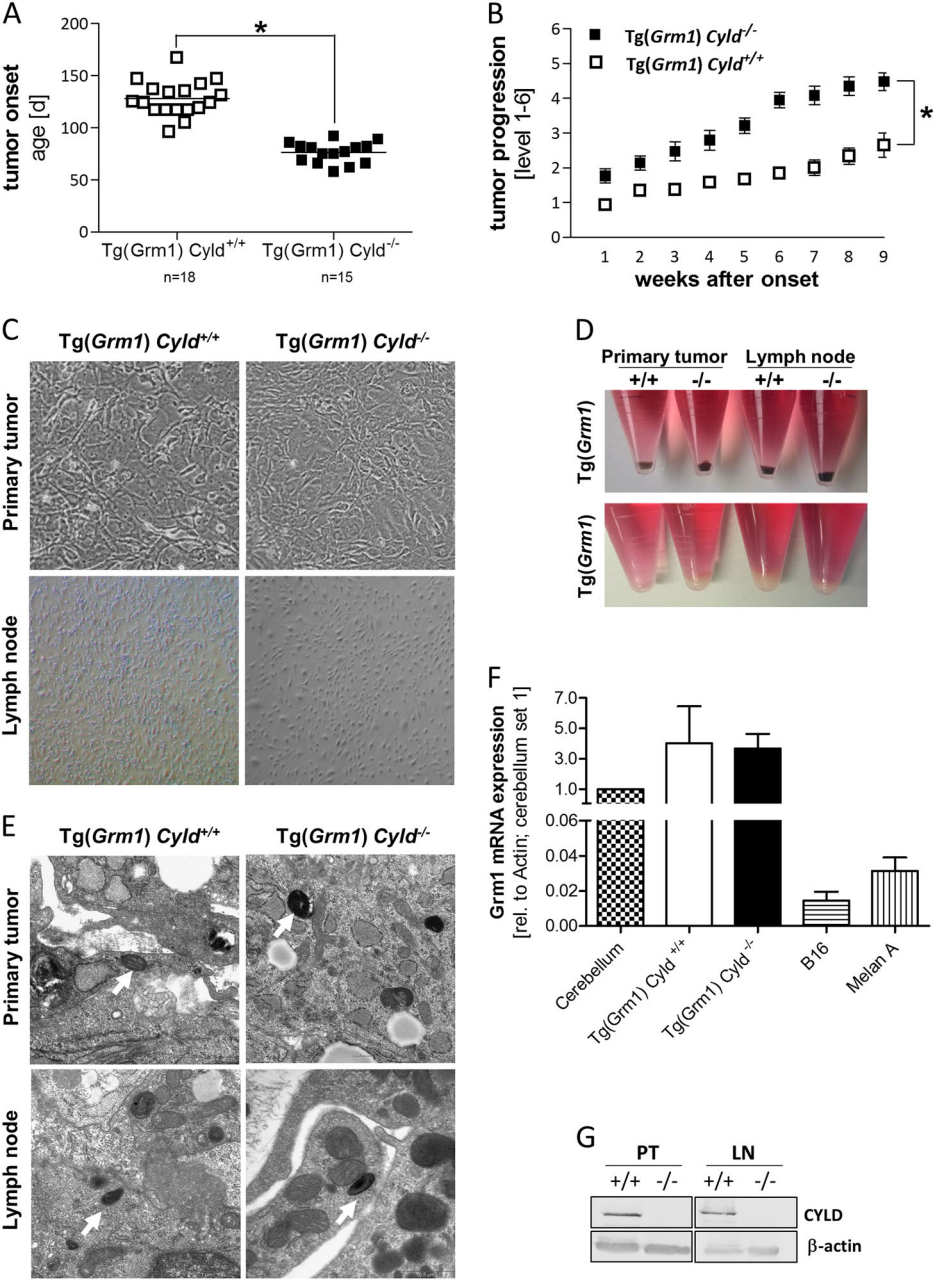
Melanoma onset and progression in vivo and generation of Tg(*Grm1*) melanoma cell lines. **a** Melanoma onset in Tg(*Grm1*) *Cyld*^*−/−*^ (*n* = 15) and Tg(*Grm1*) *Cyld*^*+/+*^ mice (*n* = 18). **b** Tumor progression of the *Cyld*-knockout mice compared with *Cyld*-wildtype mice after tumor onset. The grading system to evaluate the progression of tumor growth at the tail, ear and perianal region for additional nine weeks after tumor onset has been applied as previously described^19^. **c** For cultivation of murine melanoma cell lines tissue of primary melanoma (ear and tail), as well as metastatic melanoma tissue (lymph node) was taken from Tg(*Grm1*) *Cyld*^*+/+*^ and Tg(*Grm1*) *Cyld*^*−/−*^ mice. **d** Loss of pigmentation of the cell lines was observed after a few passages. **e** Transmission electron microscopy analysis of spheroids from primary tumor cell line and metastatic cell line gained from *Cyld*^*+/+*^ and *Cyld*^*−/−*^ mice displayed melanosomes (arrow). **f** Quantification of *Grm1* mRNA expression of the generated Tg(*Grm1*) cell lines (*n* = 10) compared with murine cerebellum as control (set as 1) was measured via qRT-PCR analysis. MelanA and B16 were used as negative control and β-Actin served as reference gene. **g** CYLD protein level was measured via western blot to confirm the *Cyld* genotype. GAPDH was used as loading control. (*p < 0.05)

To study the role of CYLD in melanoma tumorigenesis in vivo, Tg(*Grm1*) mice^11^ were crossbred with C57BL/6 *Cyld*-knockout mice^19^. The generated Tg(*Grm1*) *Cyld*^*+/+*^ and Tg(*Grm1*) *Cyld*^*−/−*^ mice were then analyzed for melanoma onset (Fig. 1a). Tg(*Grm1*) *Cyld*^*−/−*^ mice develop melanoma significantly earlier compared to the Tg(*Grm1*) *Cyld*^*+/+*^control group. *Cyld*^*+/+*^ mice exhibited tumors 18 weeks after birth, whereas melanoma onset of *Cyld*^*−/−*^ mice was already observed after 10 weeks. Further, the progression of melanoma growth on ear, tail and anus were followed for nine weeks after tumor onset. Here, a scoring from minimal^1^ to extreme tumor growth^6^ was used to quantify melanoma progression as described previously^20^. This documentation revealed that *Cyld*-knockout mice display increased tumor growth compared to *Cyld*-wildtype mice (Fig. 1b). Additionally, the Grm1 mRNA expression in lymph node tissues of Tg(*Grm1*) *Cyld*^*+/+*^ and Tg(*Grm1*) *Cyld*^*−/−*^ mice was analyzed as marker for melanoma cell dissemination. Here, a significantly enhanced Grm1 expression at the age of 77d was observed in lymph nodes of the *Cyld*^*−/−*^ mice whereas no melanoma cells were detected in C*yld*^*+/+*^ mice (Supplementary Fig. S1).

### RNA sequencing revealed CYLD-dependent gene regulation

In order to define CYLD-dependent deregulated genes and the underlying mechanism resulting in increased tumor growth in the *Cyld*-knockout mice, RNA sequencing was performed^21^. Here, primary tumor tissue from Tg(*Grm1*) *Cyld*^*+/+*^ versus Tg(*Grm1*) *Cyld*^*−/−*^ was used to determine the gene expression profile (*p*-value < 0.05; 0.66 < FC > 1.5; FC = foldchange). RNA sequencing revealed 398 upregulated and 101 downregulated genes in the *Cyld-*knockdown tissue compared to the wild-type tissue (data not shown). To identify an impact of deregulated genes on pathway regulation, the differentially expressed genes were analyzed in STRING and classified corresponding to GO terms. The classification of KEGG pathways revealed many pathways known to be deregulated in cancer, for example TGF-beta and Rap1 (Table 1). Here, we confirmed the upregulation of TGF-beta signaling in *Cyld*-knockout compared to *Cyld*-wildtype cell lines using Smad2/3-responsive reporter gene assays (Supplementary Fig. S2). This is in agreement with the differential expression of several TGF-beta target genes like BMP2K or SMURF1. Interestingly, the classification according to biological processes yields regulation of cell proliferation, migration and also angiogenesis (Table 2).

**Table 1:** KEGG pathway analysis via String database (*p*-value < 0,05; 0,66 < FC^a^ >1.5)

KEGG pathway description	Observed gene count	False discovery rate	Matching proteins in the network
ECM-receptor interaction	11	7.92E−04	Col11a1,Col27a1,Col4a6,Gp1bb,Itga2b,Itgb4,Lamb3,Sdc4,Thbs2,Thbs3,Tnxb
Hedgehog signaling pathway	8	1.23E−03	Bmp2,Bmp4,Gas1,Gli1,Wnt10a,Wnt3,Wnt4,Wnt7b
Retinol metabolism	9	4.55E−03	Adh1,Aldh1a7,Cyp1a1,Cyp2b10,Cyp2b19,Rdh12,Rdh16,Sdr16c5,Ugt1a1
TGF-beta signaling pathway	9	4.55E−03	Bmp2,Bmp4,Bmp7,Dcn,Fst,Id1,Id3,Id4,Nog
Hippo signaling pathway	12	5.13E−03	Bmp2,Bmp4,Bmp7,Id1,Lats2,Nkd1,Pard6b,Trp73,Wnt10a,Wnt3,Wnt4,Wnt7b
Tight junction	11	6.66E−03	Cgn,Cldn1,Cldn23,Cldn4,Crb3,Epb4.1l3,Gnai1,Myh14,Ocln,Pard6b,Tjp3
Basal cell carcinoma	7	6.66E−03	Bmp2,Bmp4,Gli1,Wnt10a,Wnt3,Wnt4,Wnt7b
Leukocyte transendothelial migration	10	9.90E−03	Arhgap5,Cldn1,Cldn23,Cldn4,Cxcl12,Gnai1,Mapk13,Msn,Ocln,Vcl
Steroid hormone biosynthesis	8	9.98E−03	Akr1c18,Cyp1a1,Cyp2b10,Cyp2b19,Cyp7b1,Hsd17b2,Hsd3b6,Ugt1a1
Histidine metabolism	5	9.98E−03	Aldh3a1,Aldh3b2,Aspa,Hal,Maob
Metabolism of xenobiotics by cytochrome P450	7	9.98E−03	Adh1,Aldh3a1,Aldh3b2,Cbr2,Cyp1a1,Cyp2f2,Ugt1a1
Arachidonic acid metabolism	8	1.21E−02	Alox12e,Cbr2,Cyp2b10,Cyp2b19,Cyp4f18,Fam213b,Ggt6,Ptgs1
Protein digestion and absorption	8	1.41E−02	Atp1a2,Col11a1,Col17a1,Col27a1,Col4a6,Cpa3,Fxyd2,Slc15a1
Amoebiasis	9	2.02E−02	Col11a1,Col27a1,Col4a6,Gna14,Il1r2,Lamb3,Serpinb13,Serpinb2,Vcl
Rap1 signaling pathway	12	3.60E−02	Adcy7,Efna3,Efna4,Fgfr2,Fgfr3,Gnai1,Id1,Itga2b,Lpar3,Mapk13,Pard6b,Rap1gap

^a^foldchange

**Table 2:** GO analysis via String database

Term (GO number)	Observed gene count	False discovery rate
Regulation of cell proliferation (0042127)	64	4.32E−08
Regulation of cell migration (0030334)	28	1.39E−03
Regulation of angiogenesis		
(0045765)	12	2.47E−02

### Generation of Tg(*Grm1*) melanoma cell lines

On the basis of the RNA sequencing results and to gain further insight into how CYLD acts as tumor suppressor in malignant melanoma, cell lines of both Tg(*Grm1*) *Cyld*-wildtype and Tg(*Grm1*) *Cyld*-knockout mice were generated. For this purpose, melanoma cells of primary melanoma tissue from tail and ear, as well as metastatic melanoma cells from the lymph node were isolated (Fig. 1c). As pigmentation was lost after a few passages in all cell lines (Fig. 1d), the melanocytic origin was confirmed by electron microscopy showing melanosomes in all tested cell lines (Fig. 1e). In addition, qRT-PCR analyses revealed a comparable *Grm1* mRNA expression level of cultivated Tg(*Grm1*) cell lines to murine cerebellum (positive control, set as 1), whereas the murine B16 melanoma and melanocytic Melan-A cell line (negative control) displayed extremely low Grm1 expression (Fig. 1f). Moreover, GRM1 expression in the *Cyld*-wildtype and *Cyld*-knockout cell lines was comparable. This hints at the fact that the generated cells, with same genetic GRM1 background, carry and express the *Grm1* transgene controlled by the *Dct* promoter and therefore are of melanocytic origin. Furthermore, CYLD protein levels were confirmed by Western blot showing CYLD expression in cell lines gained from *Cyld*-wildtype mice and no protein in *Cyld*-knockout mice (Fig. 1g).

### *Cyld*-deficiency enhances proliferation, colony formation and migration

**Fig. 2:**
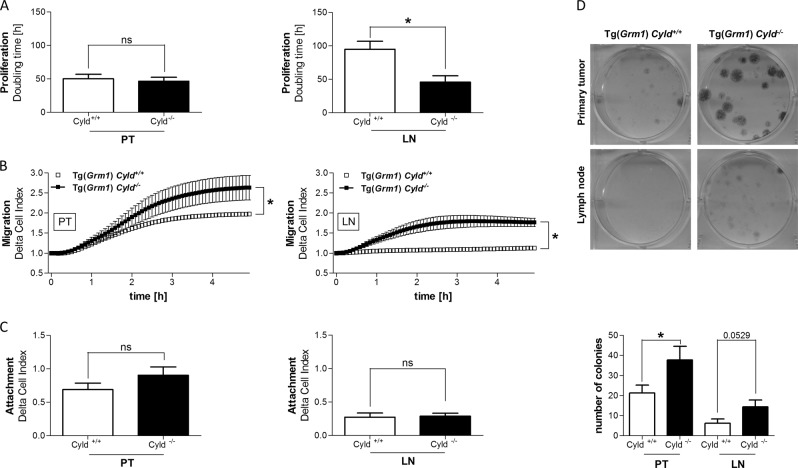
Proliferation and migration potential. **a**–**c** Proliferation **a**, migration **b** and attachment **c** analyses were performed using the xCELLigence system of Tg(*Grm1*) *Cyld*^*+/+*^ and Tg(*Grm1*) *Cyld*^*−/−*^ for cells from primary melanoma tissue (PT) and from metastatic lymph node (LN) tissue (Cell index = relative change in measured impedance to represent cell status). **d** Representative images of each one tail and lymph node cell line from both genotypes in clone-forming analyses are shown as well as the quantification. (*p < 0.05)

After generating the melanoma cell lines out of tumors from Tg(*Grm1*) *Cyld*-wildtype and Tg(*Grm1*) *Cyld*-knockout mice, the cell lines were characterized using functional in vitro assays. First, proliferation was measured by in vitro real-time proliferation assay using the xCELLigence system. This analysis revealed that the metastatic Tg(*Grm1*) *Cyld*^*−/−*^ cells show a reduced doubling time in comparison to *Cyld*-expressing cells, whereas the proliferative potential from primary tumor cells with or without CYLD expression was comparable (Fig. 2a). Because of the high metastatic tendency of melanoma cells, also the migratory potential was analyzed. Both, primary tumor and metastatic Tg(*Grm1*) *Cyld*^*−/−*^ cells display significantly increased migration compared to the cell lines derived from Tg(*Grm1*) *Cyld*^*+/+*^ *mice* (Fig. 2b), whereas cell attachment was not influenced by CYLD (Fig. 2c). Additionally, analyses of the clonogenic potential revealed an increased ability to form colonies from single cells of both primary tumor and metastatic *Cyld*-deficient Tg(*Grm1*) cells compared to *Cyld*^*+/+*^ cells (Fig. 2d).

### Loss of CYLD increases vasculogenic mimicry and promotes (lymph-) angiogenesis

**Fig. 3:**
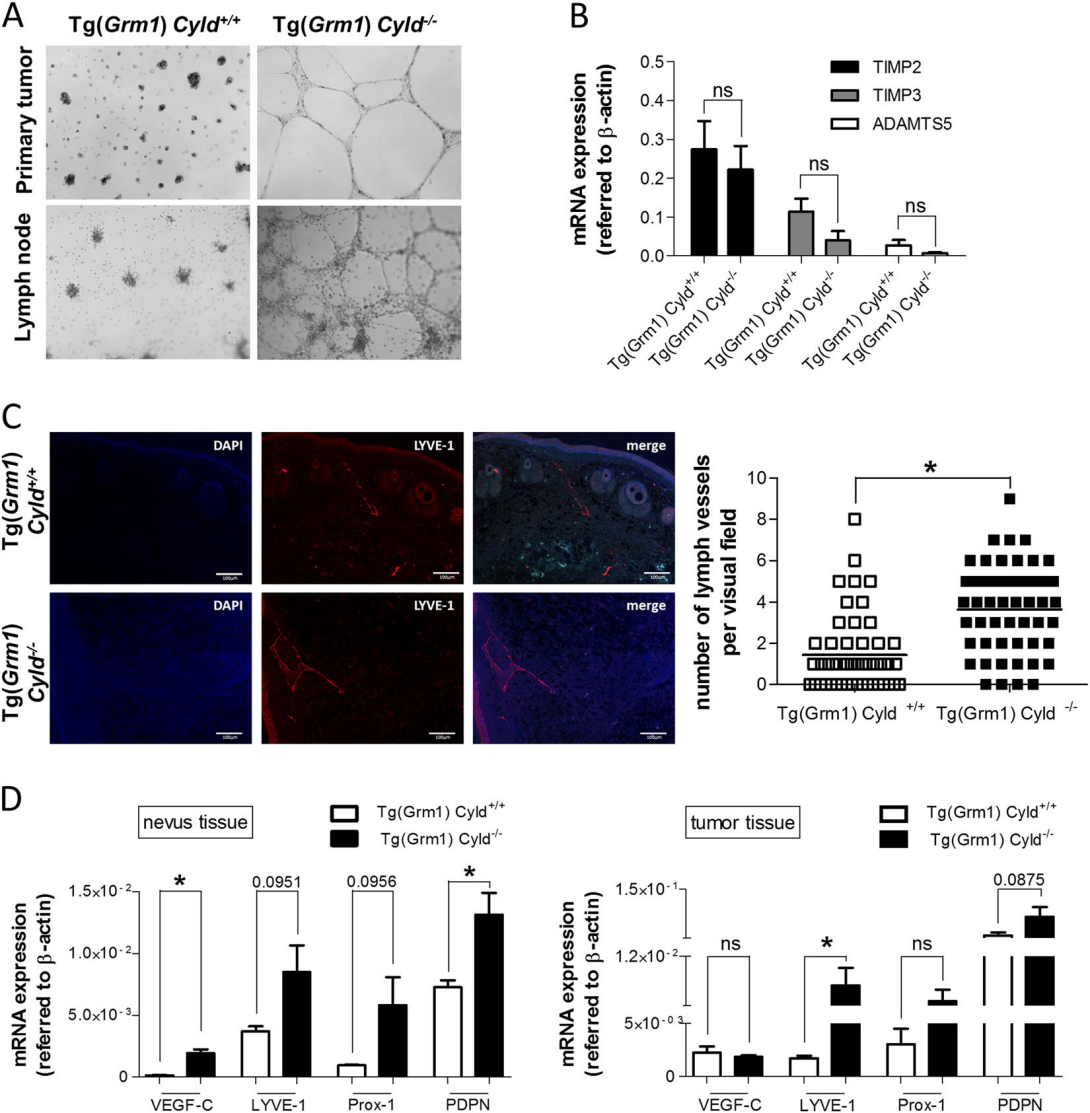
CYLD loss enhances vasculogenic mimicry and (lymph-) angiogenesis. **a** Tube formation assays reveal an enhanced ability to form vascular structures in *Cyld*^*−/−*^ cells. **b** Via qRT-PCR analyses decreased mRNA expression of anti-angiogenic markers Adamts5, Timp2 and Timp3 was detected in *Cyld*-deficient cell lines compared to *Cyld*-wildtype cells. **c** LYVE-1 (red) immunofluorescence staining of Tg(*Grm1*) *Cyld*^*+/+*^ and Tg(*Grm1*) *Cyld*^*−/−*^ melanoma tail tissue. DAPI (blue) were used for nuclear staining. For quantification the number of lymphatic vessels was counted manually per visual field. **d** mRNA expression of lymph angiogenesis marker in nevus and tumor tissue. (*p < 0.05)

As vasculogenic mimicry is a well-known feature of melanoma, the impact of CYLD on melanoma cells to form vascular tubes was analyzed^22,23^. Tube formation assays were performed with the murine cell lines from primary melanoma and melanoma metastasis (Fig. 3a). All Tg(*Grm1*) *Cyld*^*−/−*^ cell lines were able to build vascular structures, whereas only one of six Tg(*Grm1*) *Cyld*^*+/+*^ cell lines showed this ability. To characterize this interesting difference in more detail, we studied the influence of CYLD-deficiency on angiogenesis. Studies show that the expression of *Timp2* (*tissue inhibitor of metalloproteinase 2*), *Timp3* (tissue inhibitor of metalloproteinase 3) and *Adamts5* (*a disintegrin-like and metalloproteinase with thrombospondin type 1 motif, 5*) reduce angiogenesis in melanoma cells^24–26^. Therefore, mRNA expression patterns of these anti-angiogenic markers were examined. Although differences are not significant, Tg(*Grm1*) *Cyld*^*−/−*^ cell lines display a weaker expression of each marker compared to Tg(*Grm1*) *Cyld*^*+/+*^ cells (Fig. 3b).

Besides blood vessel formation, especially lymph angiogenesis is of particular importance in melanoma progression. As melanoma cells metastasize predominantly via lymphatic vessels into lymph nodes or distant organs, we aimed at understanding the relevant factors modulated by CYLD in this process. First, we investigated the role of CYLD in the formation of lymphatic vessels via immunofluorescence staining on murine Tg(*Grm1*) *Cyld*^*+/+*^ and *Cyld*^*−/−*^ melanoma tissues using LYVE-1, a specific lymphatic endothelial marker (Fig. 3c). Quantitative analyses revealed a markedly higher number of lymphatic vessels in melanoma tissues of *Cyld*-deficient mice compared to *Cyld*-wildtype mice indicating that CYLD may represent a regulator in melanoma lymph angiogenesis. Quantitative RT-PCR analyses were used to examine the mRNA expression pattern of well-known lymph angiogenetic markers in nevus and tumor tissue of the model (Fig. 3d). This revealed an increased mRNA expression of VEGF-C, Podoplanin, LYVE-1 and Prox-1 in the nevus tissues *Cyld*-deficient mice. Although in tumor tissues only LYVE-1 expression is significantly upregulated, there is a notable tendency of higher expression of nearly all markers after loss of CYLD, which supports the role of CYLD in regulating lymph angiogenesis.

## Discussion

In this study, a mouse model was newly developed to investigate the impact of CYLD deficiency on melanoma. The model was generated on the background of the Tg(Grm1) melanoma mouse model showing spontaneous melanoma development^11^. CYLD is known to have a tumor-suppressive role in human melanoma. Previous studies, including studies from our group, were able to link loss of CYLD to increased proliferation and invasion in vitro and tumor progression in vivo^5,7^. However, its role in melanoma migration seems to be contradictory. Whereas some publications reported a diminished migratory potential upon *CYLD* re-expression in human melanoma cell lines^5,7^, one study described a reduced migration when CYLD-expressing melanoma cells were treated with siRNA against CYLD^6^. Moreover, the function of CYLD in metastasis is poorly examined and related processes, as lymph angiogenesis, were not analyzed previously.

Consistent with the strong differences in melanoma development in the *Cyld*-deficient and wildtype mice, RNA sequencing analysis revealed many deregulated genes in *Cyld*-deficient melanoma tissue. A study from Lim et al. demonstrated that CYLD acts as a negative regulator via inhibition of TGF-β signaling in fibrosis^27^. The connection between CYLD and TGF-β signaling, observed by STRING analyses of the RNA-seq data, was confirmed by showing higher TGF-β pathway activity and increased expression of SMAD target genes (e.g. Bmp2k and Smurf1) in *Cyld*-knockout versus *Cyld*-wildtype cells and tissues. STRING analyses further confirmed the relevance of CYLD in the regulation of proliferation and migration as it was described already in human melanoma^5^. However interestingly, it also provided the first hints of an important role of CYLD in angiogenesis, the formation of new blood vessels from preexisting ones.

In line with the RNA sequencing data, in vitro studies with newly generated Tg(Grm1) melanoma cell lines displayed an enhanced proliferation, migration and clonogenicity in *Cyld*-knockout cell lines. These data are in agreement with previously published results gained from human melanoma cell lines and also demonstrate that the generated cell lines are a valid model for future studies of the tumor suppressor functions of CYLD^5,7^.

Angiogenesis is a well-studied process in melanoma that helps the tumor to ensure the supply with nutrients and oxygen and promotes metastasis. Several angiogenic serum factors are elevated in melanoma patients^28^ and high rate of angiogenesis correlates with poor prognosis for melanoma patients. Melanoma cells are known to have the intrinsic capacity of vascular mimicry^29^. In this study, we revealed that CYLD has a regulatory role in vasculogenic mimicry of melanoma cells by suppressing tubular formation. Contradictory, a study from Gao et al. showed that the knockdown of CYLD expression impairs endothelial tube formation and sprouting of endothelial cells^30^. This difference hints to a specific function of CYLD in different cell types and supports a role of CYLD in vascular mimicry of melanoma cells.

Our data indicate that loss of CYLD contributes to angiogenesis in melanoma by a potential negative effect on expression of several anti-angiogenic factors like TIMP-2, TIMP-3 and ADAMTS5. TIMP3 is described as an antagonist of VEGF-mediated angiogenesis^25^ and ADAMTS5 was found to inhibit endothelial cell tube formation^31^. TIMP2 was shown to reduce the tube formation ability of the murine melanoma cell line B16^24^. Moreover, in esophageal carcinoma TIMP-2 expression is negatively correlated with lymph node metastasis via inhibition of MMPs^32^.

Interestingly, the re-expression of a catalytically inactive form of CYLD (CYLD^C/S^) compared to the wildtype CYLD re-expression did not result in decreased level of angiogenic factors, including VEGF-A^33^. Moreover, only the overexpression of CYLD in comparison to CYLD^C/S^ reduced angiogenesis in human squamous cell carcinomas^34^. Therefore, it seems that the deubiquitinase function of CYLD is important for the regulation of angiogenesis. This is supported by a study showing that CYLD acts in the process of transdifferentiation of adventitial fibroblasts via its deubiquitinase function and thus plays a role in vascular remodeling^35^.

We, further, asked whether CYLD also influences the formation of lymphatic structures. In previous studies, the marker LYVE-1 was established to show that a high number of lymphatic vessels is prognostic for lymph node metastasis and metastasis in general. Further, an increased lymph vessel quantity is associated with poor outcome^36–38^. Interestingly, melanoma tissue of *Cyld*-knockout mice exhibits more LYVE-1 positive vessels than the control group, thus CYLD is involved in the process of lymph angiogenesis. This is further corroborated by the observation that *Cyld*-deficiency enhances the expression of several established lymph angiogenesis markers^39^. Remarkably, in our study already the benign nevus tissue of *Cyld*-deficient mice showed significantly enhanced expression of almost all investigated markers. Based on our results, loss of CYLD positively affects the formation of lymph vessels in melanoma and enhances metastasis, supporting the important role of CYLD especially in the early process of melanoma progression. In the context of angiogenesis and lymph angiogenesis, the analyses of the RNA sequencing data via STRING further supported the role of CYLD in these processes. Several cancer related pathways and processes differentially regulated in the sequencing data are known for their relevance in the formation of blood and lymph vessels. Here, also the in vitro data provided strong evidence that *Cyld*-deficiency promotes expression of several genes and signaling pathways that were already described to be involved in the process of angiogenesis as well as in lymph angiogenesis. All these findings support our hypothesis of the important role of CYLD in lymph-/angiogenesis.

In summary, the newly generated mouse model and the resulting cell lines of primary tumors and metastases represent a good base for studies of the function of CYLD in malignant melanoma. Here, a novel function for CYLD in both vasculogenic mimicry and lymph angiogenesis in melanoma was characterized. Furthermore, our findings demonstrate the crucial impact of CYLD on melanoma onset, progression and metastasis, as well as its multiple tumor-suppressive functions.

## Material & methods

### Mice

The transgenic Tg(*Grm1*) mice^11^ were established at the Department of Chemical Biology, Rutgers University, Piscataway, USA and provided by Prof. Chen and Prof. Becker. C57Bl/6 J *Cyld*-knockout mice^19^ were provided by Dr. Massoumi (Department of Laboratory Medicine, Lund University, Sweden). Mice were kept under standard conditions at 21 °C ( ± 1 °C) with 55% ( ± 10%) relative humidity and 12 h light/dark intervals. Animals were fed with standard chow (Ssniff, Soest, Germany) and with drinking water *ad libitum*. Animal care and experimental procedures were carried out in accordance with the guidelines of the German law governing animal care. Experiments were approved by the Ethics Committee for Animal Research of the Bavarian government. For all analyses, homozygous transgenic Tg(*Grm1*) *Cyld*^*+/+*^ and Tg(*Grm1*) *Cyld*^*−/−*^ animals (litter mates) were used. For analyzing metastasis in the lymph nodes, mRNA from lymph node tissues was isolated as previously described and qRT-PCR for Grm1 normalized on ß-actin was performed^40^.

### Cell culture

Tg(*Grm1*) melanoma cell lines were generated, as previously described^14^. They were cultivated in DMEM containing 4500 mg glucose/l, 110 mg sodium pyruvate/l and l-glutamine and supplemented with 10% fetal bovine serum, amphotericin B (2.5 µg/ml) and 5% penicillin/streptomycin (all from Sigma-Aldrich, München, Germany). Cell lines were incubated in a humidified atmosphere containing 8% CO_2_ at 37 °C. Growth medium was renewed twice a week and cells were spilt at a ratio 1:3 to 1:5 once weekly.

### Clonogenic assay

For clonogenic forming assays 250 cells were seeded in a well of a six-well plate. After an incubation time of ten days at 37 °C and 8% CO_2_ cells were rinsed with PBS. Subsequently, colonies were fixed with 6% v/v glutaraldehyde and stained with crystal violet as described elsewhere^41^. Quantity of colonies was determined manually.

### Cell migration and proliferation assays

In order to study the migratory and proliferative potential of Tg(*Grm1*) cells, the xCELLigence System was used (Roche, Mannheim, Germany)^14,42–44^. This instrument monitors the behavior of the cells in real time by measuring the electrical impedance across interdigitated microelectrodes covering the bottom of the plates^43^. The software calculates the cell index, a relative and dimensionless parameter of the electrode impedance to represent cell status. For migration analysis 2 × 10^4^ cells in 100 μl DMEM medium without FCS were seeded into the upper chambers of CIM-plates (similar to Boyden chamber) and were measured for 8 h. For attachment and proliferation experiments 2 × 10^3^ and 4 × 10^3^ cells in 100 μl medium were seeded into E-Plates and electrical impedance was measured during 4 h and 90 h, respectively. Each cell line was measured in duplicate in two independent experiments.

### Matrigel tube formation assay

For tube formation assays 200 µl matrigel was added into each well of a eight-chamber culture slide (both from BD Biosciences, Bedford, USA). After polymerization at 37 °C murine Tg(*Grm1*) melanoma cells were seeded into each well in a total volume of 400 µl DMEM high glucose medium (Sigma-Aldrich, Munich, Germany). Tube formation was photographically documented after 16 h incubation at 37 °C using an IX83 microscope (Olympus, Tokyo, Japan).

### Transmission electron microscopy

For electron microscopy Tg(*Grm1*) cells were prepared as follows: 8 × 10^3^ cells were seeded in 100 µl medium in one well of a 96-well plate coated with 1% agarose to form spheroids. After an incubation time of three days at 37 °C spheroids were rinsed with PBS and fixed in 2.5% glutaraldehyde for 2 h at 4 °C. After a further washing step with PBS spheroids were postfixed in osmium tetroxide, embedded in Epon according to standard protocols and ultrathin sections were examined in a Zeiss 906 electron microscope (Zeiss, Oberkochen, Germany).

### RNA isolation, Reverse Transcription and Quantitative RT-PCR

Total RNA was isolated using the E.Z.N.A. MicroElute Total RNA Kit (Omega Bio-Tek, VWR Darmstadt, Germany) according to the manufacturer’s instructions. RNA concentration was measured with a NanoDrop spectrophotometer (Peqlab Biotechnology GmbH, Erlangen, Germany) and cDNA was generated by reverse transcription using the Super Script II Reverse Transcriptase Kit (Life Technologies, Carlsbad, USA) with each reaction containing 500 ng of total RNA. Analysis of mRNA expression was performed using quantitative Real-Time PCR on the LightCycler 480 system (Roche, Mannheim, Germany). A volume of 1 μl cDNA template, 0.5 μl of forward and reverse primers (each 20 μM) and 10 μl of SYBR Green I (Roche, Mannheim, Germany) were combined to a total volume of 20 μl. The following primers were used: Adamts5 for 5′-GCCATTGTAATAACCCTGCACC-3′; Adamts5 rev 5′-TCAGTCCCATCCGTAACCTTTG-3′; β-Actin for 5′-TGGAATCCTGTGGCATCCATGAAAC-3′; β-Actin rev 5′-TAAAACGCAGCTCAGTAACAGTCCG-3′; Grm1 for 5′-GGGCAGGGAACGCCAATTCT-3′; Grm1 rev 5′-TGGAAGGGCTGCTGGGAGGG-3′; Timp2 for 5′-GCAGACGTAGTGATCAGAGCC-3′; Timp2 rev 5′-TCCCAGGGCACAATGAAGTC-3′; Lyve-1 for 5′-ACAGTGTGACATTTGCCCCT-3′; Lyve-1 rev 5′-CAGCCCACACTCCGCTATAC-3′; PDPN for 5′-GTGCTACTGGAGGGCTTAATGA-3′; PDPN rev 5′-TGTTGTCTGCGTTTCATCCCC-3′; Prox-1 for 5′-GAGATGTGTGAGCTGGACCC-3′; Prox-1 rev 5′-CCTGAGGAACCTGGCGAGAG-3′; VEGF-C for 5′-CTTCTTGTCTCTGGCGTGTTC-3′; VEGF-C rev 5′-GGTACAGGACAGACATCAGCTC-3′; Timp3 for 5′-GACCCTTGGCCACTTAGTCC-3′ and Timp3 rev 5′CGGATCACGATGTCGGAGTTG-3′. Each sample was analyzed in duplicates. The target cDNA was normalized to β-Actin levels.

### Protein isolation and Western blotting

Cells were lysed in 200 µl RIPA buffer (Roche, Mannheim, Germany) for 15 min on 4 °C and thereafter the cell debris was separated via centrifugation at 13,000 rpm and 4 °C for 10 min. Protein concentration was determined using the Pierce BCA Protein Assay Kit (Thermo Fisher Scientific, Rockford, USA). In total 40 µg of total lysate per lane were separated on 10% SDS–PAGE gels and subsequently transferred onto a PVDF membrane. After blocking for 1 h with 5% BSA/TBS-T the membrane was incubated overnight (4 °C) with one of the following antibodies: anti-GAPDH (Cell Signaling Technology, Frankfurt a.M.; 1:1000) or CYLD (Cell Signaling Technology, Frankfurt a.M.; 1:1000). After washing the membrane three times with TBS-T an incubation step followed for 1 h with the alkaline phosphate-coupled secondary antibody anti-rabbit AP (Cell Signaling Technology, 1:4000). Finally, the membrane was washed again for three times in TBS-T and the immunoreaction was then visualized using NBT/BCIP (Life technologies, Carlsbad, USA).

### Immunofluorescence

For immunofluorescence staining of tissue samples 5 µm sections of formalin-fixed and paraffin-embedded tissue blocks were used and treated, as described previously^14^. The incubation with the anti-LYVE-1 antibody (Abcam, Cambridge; 1:100 in 2% BSA/TBS) occurred over night at 4 °C and after three washing steps with PBS the secondary antibody Alexa fluor 488 anti-rabbit (Life technologies, Carlsbad, USA; 1:500 in PBS) was added for 1 h at 37 °C. Finally, tissue sections were washed again with PBS and VECTASHIELD^TM^ Slide Mounting Medium with DAPI (Vector Laboratories Inc., Burlingame, USA) was added for mounting. Photographic documentation was performed using the Axio Imager Zeiss Z1 microscope (Axiovision, Carl Zeiss, Oberkochen, Germany).

### Luciferase reporter gene assay

The tumor cell lines (150,000 cells/well) were seeded into six-well plates and transiently transfected with 1 μg plasmids (p(CAGA)_9_ LUC^45^, pGL2basic) using the lipofectamine plus method (Invitrogen) according to the manufacturer’s instructions. For normalization of the transfection efficiency 0.1 μg of a pRL-TK plasmid (Promega, Mannheim, Germany) was co-transfected. After 24 h of transfection the cells were harvested. The lysate was analyzed for luciferase activity with a luminometer using Promega dual-luciferase assay reagent^46^.

### RNA sequencing

Sample processing was performed at an Affymetrix Service Provider and Core Facility, “KFB - Center of Excellence for Fluorescent Bioanalytics” (Regensburg, Germany; www.kfb-regensburg.de)^21^. For the identification of known and predicted protein–protein interactions, STRING (Search Tool for the Retrieval of Interacting Genes/Proteins) database was used^47^.

### Statistical analysis

Results are shown as the mean ± standard error of the mean or percent and statistical significance was determined using the Student’s unpaired *t*-test calculated with the software GraphPad Prism (GraphPad Software, Inc., San Diego, USA). A *p*-value < 0.05 was considered as statistically significant (ns: not significant, *p < 0.05).

## Supplementary information

Supplementary Figure
