# Supplementary material for: Loss of CYLD accelerates melanoma development and progression in the Tg(Grm1) melanoma mouse model
Source: Oncogenesis. 2019 Oct 7;8(10):56. doi: 10.1038/s41389-019-0169-4 (PMC6779913; doi:10.1038/s41389-019-0169-4)
Supplement: Supplementary file 1 — Supplementary Figure [file 41389_2019_169_MOESM1_ESM.pdf]

Supplementary Figure 1

A

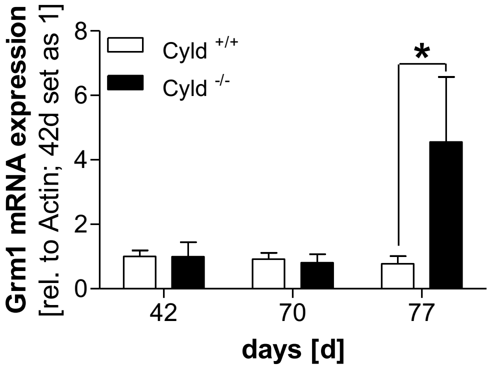

Supplementary Fig.S1: Development of lymph node metastasis in Tg(*Grm1*) *Cyld wt* and *deficient mice*

Grm1 mRNA expression level of lymph node tissues from *Cyld*-wildtype and *Cyld*-knockout mice analyzed at thee age of day 42, 70 and 77 of the mice (42d set1). (*: $p < 0.05$)

Supplementary Figure 2

A

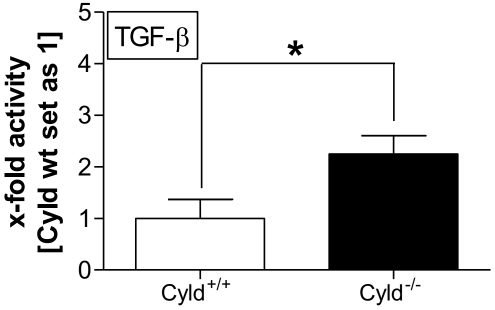

Supplementary Fig.S2: Analysis of TGF-beta signaling

Luciferase reporter gene assay using $(CAGA)_9$LUC vector revealed a significant enhanced TGF-β activity of the *Cyld*-knockout compared to *Cyld*-wildtype cell line. (*: $p < 0.05$)
